# Supplementary material for: Colorimetric Sensor Based on Hydroxypropyl Cellulose for Wide Temperature Sensing Range
Source: Sensors (Basel). 2022 Jan 24;22(3):886. doi: 10.3390/s22030886 (PMC8839604; doi:10.3390/s22030886)
Supplement: Supplementary file 1 [file sensors-22-00886-s001.zip › sensors-1507293-supplementary.pdf]

Supplementary materials

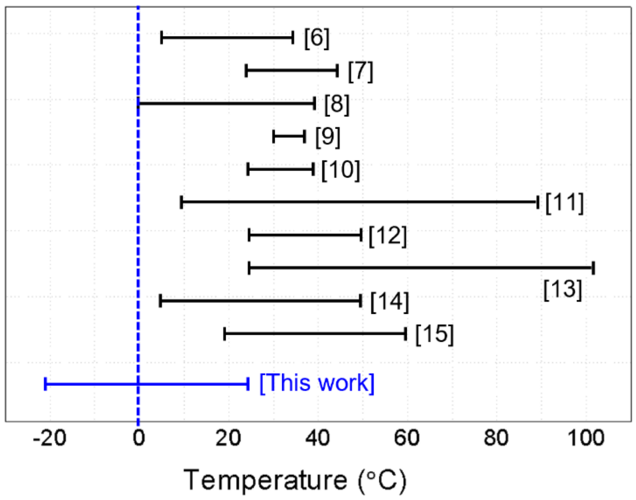

Figure S1. Temperature sensing ranges of various colorimetric sensors.

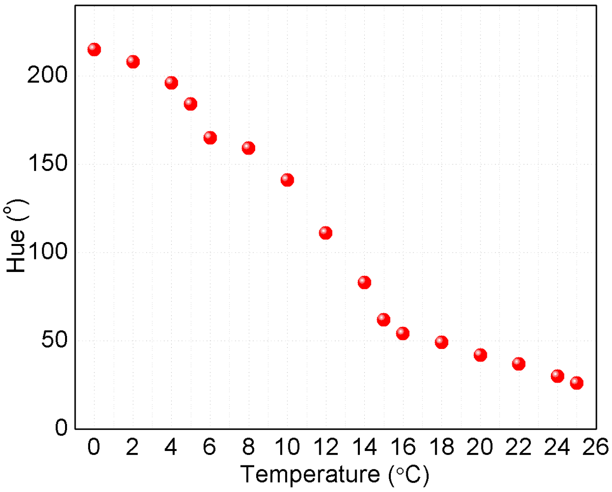

Figure S2. Color response of the HPC colorimetric sensor as a function of temperature depicte d in the HSV color space.

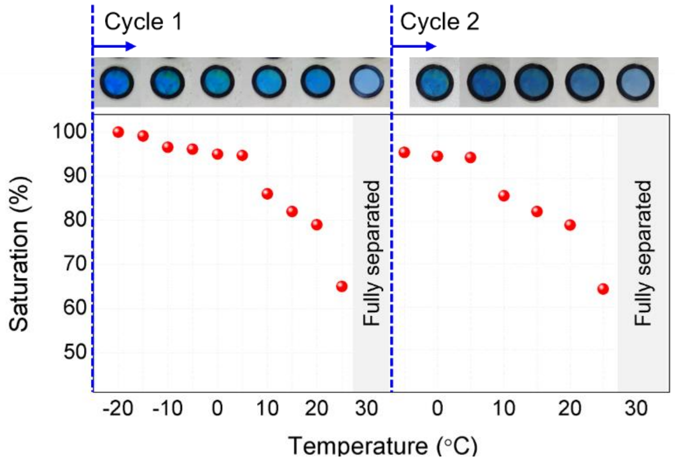

Figure S3. Repeatability test of the ethylene glycol-modulated (30 wt.%) HPC colorimetric sens -or.
